# Cardiovascular risk in primary aldosteronism: inflammatory drivers, residual risk, and emerging combination strategies

**DOI:** 10.3389/fendo.2026.1802307

**Published:** 2026-04-15

**Authors:** Haoze Liu, Zejun Wu, Xinzhe Liu, Yuyuan Hu, Kaixuan Zhang, Jiwen Shang

**Affiliations:** 1 Third Hospital of Shanxi Medical University, Shanxi Bethune Hospital, Shanxi Academy of Medical Sciences Tongji Shanxi Hospital, Taiyuan, China; 2 Department of Urology, Peking University People’s Hospital, Beijing, China; 3 Department of Urology, Shanxi Bethune Hospital, Shanxi Academy of Medical Science, Tongji Shanxi Hospital, Third Hospital of Shanxi Medical University, Taiyuan, China

**Keywords:** aldosterone, aldosterone synthase inhibitors, cardiovascular risk, G protein–coupled estrogen receptor, inflammation, mineralocorticoid receptor, primary aldosteronism, residual risk

## Abstract

Primary aldosteronism (PA) is associated with a substantially higher cardiovascular risk than essential hypertension, a disparity that cannot be fully explained by blood pressure elevation alone. Clinical studies consistently demonstrate that cardiovascular morbidity and mortality often persist in patients with PA despite adequate blood pressure control and standard therapy, underscoring the existence of residual cardiovascular risk. Accumulating experimental and clinical evidence identifies inflammation as a central mediator of aldosterone-induced cardiovascular injury. Excess aldosterone drives immune–inflammatory remodeling through coordinated activation of innate and adaptive immune responses, including macrophage- and T cell–dependent pathways, as well as downstream signaling cascades such as inflammasome activation and interleukin-6–related trans-signaling. These processes promote myocardial fibrosis, vascular dysfunction, and adverse cardiac remodeling, providing a mechanistic basis for the heightened cardiovascular risk observed in PA. Although mineralocorticoid receptor (MR) antagonists remain the cornerstone of medical therapy for PA, MR blockade alone may be insufficient to fully suppress aldosterone-driven inflammatory and non-hemodynamic effects. Persistent activation of these pathways offers a plausible explanation for the residual cardiovascular risk observed in treated patients. Emerging therapeutic strategies aim to overcome these limitations through combination approaches. Aldosterone synthase inhibitors (ASIs), by targeting aldosterone production upstream, may complement MR antagonism, while interventions directed at inflammatory pathways and non-genomic aldosterone signaling could further enhance cardiovascular protection. This review integrates current mechanistic and clinical evidence on inflammatory drivers and residual risk in PA and discusses emerging combination strategies to optimize cardiovascular risk reduction in this high-risk population.

## 1 Introduction

The adult adrenal cortex is composed of the zona glomerulosa (zG), zona fasciculata (zF), and zona reticularis (zR), which predominantly synthesize mineralocorticoids, glucocorticoids, and adrenal androgens, respectively (1). Aldosterone is the principal mineralocorticoid in the human body and is synthesized and secreted by cells of the zona glomerulosa (zG) (1–3). Its biosynthesis uses cholesterol—derived from dietary intake or endogenous synthesis—as a precursor and proceeds through a series of enzymatic reactions within the mitochondria and endoplasmic reticulum (3). Unlike many other hormones, aldosterone is not stored within zona glomerulosa (zG) cells but is synthesized and released immediately in response to stimulatory signals. Aldosterone secretion is primarily regulated by angiotensin II (Ang II) and serum potassium (K^+^) concentrations, with marked increases in secretion observed when levels of Ang II or K^+^ are elevated (3–5). Adrenocorticotropic hormone (ACTH) can transiently stimulate aldosterone synthesis under stress conditions; however, its effect is rapid and short-lived (6). In addition, multiple factors, including atrial natriuretic peptide (ANP), somatostatin, dopamine, β-endorphin, acetylcholine, endothelin-1, lipoproteins, and sphingosine-1-phosphate (S1P), also contribute to its fine-tuned regulation (3, 7–9). Aldosterone is a key steroid hormone regulating salt and water homeostasis and serves as the terminal effector of the renin–angiotensin–aldosterone system (RAAS). It primarily acts on the distal tubules and collecting ducts of the kidney (also influencing the colon and salivary glands) to maintain electrolyte balance by promoting Na^+^ reabsorption and K^+^ excretion. Sustained aldosterone excess leads to Na^+^ and water retention, increased intravascular volume, and elevated blood pressure, thereby playing a central pathological role in the development and progression of hypertension (4). Against this background, the persistent dysregulation of aldosterone secretion in primary aldosteronism (PA) results in chronic exposure of the cardiovascular system to excess aldosterone, thereby establishing a pathophysiological basis for the subsequent activation of inflammatory responses and the development of cardiovascular injury.

Hypertension is one of the leading risk factors for cardiovascular morbidity and premature mortality worldwide (10). According to the 2025 American Heart Association/American College of Cardiology (AHA/ACC) guidelines for the management of adult hypertension, individuals with persistently elevated blood pressure (≥130/80 mmHg) in whom identifiable secondary causes have been excluded are generally defined as having essential hypertension (11). Compared with essential hypertension, secondary hypertension is less common overall but is often attributable to identifiable underlying causes, among which PA is one of the most prevalent (12). Multiple population-based studies consistently indicate that, among unselected hypertensive outpatient populations, the prevalence of PA is approximately 5%–10% (13, 14) and increases with the severity of hypertension (15). In patients with resistant hypertension, the prevalence can reach approximately 20% or even higher (16–18). Although hypokalemia has long been regarded as a classic feature of PA, only approximately 28% of patients exhibit overt hypokalemia (19). Consequently, screening strategies that rely on hypokalemia alone result in substantial underdiagnosis. Clinical practice also indicates that the diagnosis of PA is frequently delayed (20). By the time of diagnosis, patients often already exhibit varying degrees of end-organ damage (21), including renal injury, abnormalities in cardiac structure and function, and complications involving the central nervous system. These observations suggest that PA-related cardiovascular risk may be initiated before clinical recognition and that its development likely involves pathological mechanisms beyond hemodynamic factors alone.

Multiple case–control studies consistently demonstrate that, compared with patients with essential hypertension matched for age, sex, and blood pressure levels, individuals with PA have significantly higher rates of cardiovascular morbidity and mortality (22–26). These adverse outcomes include coronary artery disease, stroke, atrial fibrillation, left ventricular hypertrophy, heart failure, and myocardial infarction, and this elevated risk appears to be independent of the level of blood pressure control, suggesting that aldosterone excess exerts cardiovascular injury beyond hemodynamic mechanisms. For example, a meta-analysis encompassing 31 case–control studies, including a total of 3,838 patients with PA and 9,284 patients with essential hypertension, reported significantly increased risks of cardiovascular and cerebrovascular events, left ventricular hypertrophy, as well as metabolic syndrome and diabetes in the PA group (23). Moreover, among patients with hypertension and atrial fibrillation of unknown etiology, the prevalence of PA has been reported to be as high as 30%–42% (27, 28), and patients with PA or an elevated aldosterone-to-renin ratio (ARR) exhibit a higher left ventricular mass index (LVMI) (28). Collectively, these lines of evidence indicate that chronic excess exposure to aldosterone exerts substantial detrimental effects on the cardiovascular system, thereby underscoring the importance of early screening, timely diagnosis, and targeted treatment of PA.

In this review, we systematically summarize the evidence for inflammatory mechanisms underlying aldosterone-mediated cardiovascular injury in PA, explore the concept of residual cardiovascular risk beyond mineralocorticoid receptor (MR) blockade, and highlight emerging combination therapeutic strategies, including nonsteroidal mineralocorticoid receptor antagonists (MRAs), aldosterone synthase inhibitors (ASIs), and potential immunomodulatory interventions.

## 2 Overview of the integrated mechanisms of aldosterone-mediated cardiovascular injury

### 2.1 Widespread tissue distribution of the mineralocorticoid receptor provides a structural basis for the multisystem effects of aldosterone

**Figure 1:**
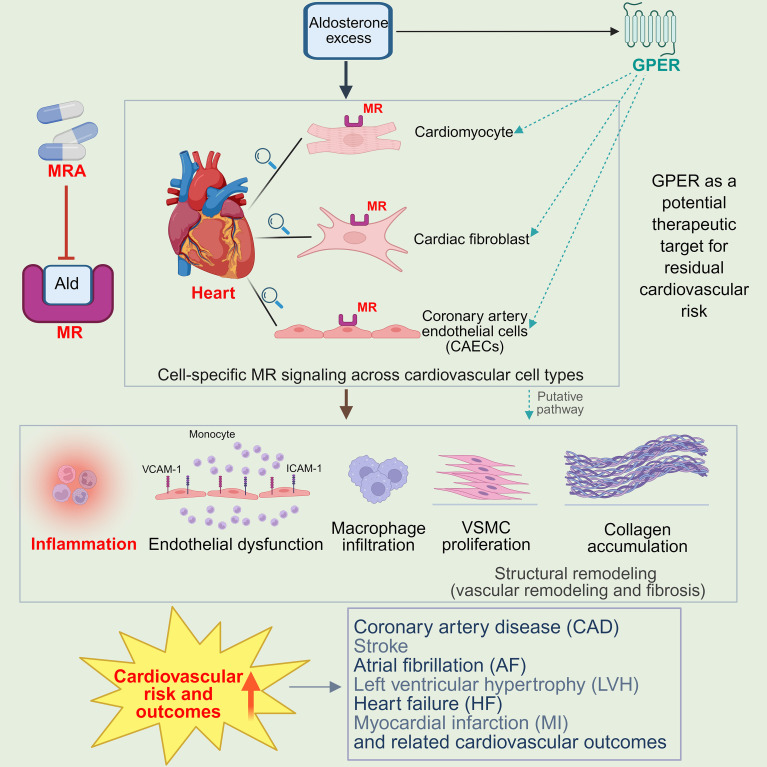
Integrated mechanisms of aldosterone-mediated cardiovascular injury and residual risk in primary aldosteronism. This schematic illustrates how aldosterone excess drives cardiovascular injury through canonical mineralocorticoid receptor (MR)–dependent pathways and non-canonical, rapid signaling mediated by the G protein–coupled estrogen receptor (GPER). Functional MR expression in cardiomyocytes, cardiac fibroblasts, and coronary artery endothelial cells (CAECs) enables cell-specific aldosterone signaling that promotes endothelial dysfunction, vascular smooth muscle cell (VSMC) proliferation, macrophage infiltration, and collagen accumulation. Although steroidal and nonsteroidal mineralocorticoid receptor antagonists (MRAs) attenuate MR-dependent inflammation and remodeling, residual cardiovascular risk may persist due to incomplete aldosterone suppression and MR-independent, GPER-associated signaling. These processes collectively contribute to adverse cardiovascular outcomes, including coronary artery disease, atrial fibrillation, stroke, left ventricular hypertrophy, heart failure, and myocardial infarction. Ald, aldosterone; MR, mineralocorticoid receptor; MRA, mineralocorticoid receptor antagonist; GPER, G protein–coupled estrogen receptor; CAECs, coronary artery endothelial cells; VSMC, vascular smooth muscle cell; CAD, coronary artery disease; AF, atrial fibrillation; LVH, left ventricular hypertrophy; HF, heart failure; MI, myocardial infarction.

Extensive experimental and clinical studies indicate that aldosterone excess is closely associated with vascular and cardiac remodeling, myocardial fibrosis, and endothelial dysfunction, thereby significantly increasing the risk of cardiovascular events and mortality (29). This effect is closely related to the widespread tissue distribution of the MR. The MR is a nuclear receptor that is essential for regulating sodium and potassium transport in epithelial cells, particularly in the renal and colonic epithelium (30, 31). Beyond epithelial tissues, studies have further demonstrated the expression of functional MR in cardiomyocytes, cardiac fibroblasts, and coronary artery endothelial cells (CAECs) (32–34). This provides a structural basis for aldosterone to exert effects across multiple cardiac cell types and helps explain the complexity of its cardiovascular actions (**Figure 1**).

### 2.2 The canonical MR pathway: genomic and non-genomic effects driving inflammation, remodeling, and fibrosis

Traditionally, aldosterone is thought to exert its effects primarily through intracellular MR–mediated genomic and non-genomic pathways (35). MR activation can induce vascular inflammatory responses (34–36) and lead to endothelial injury (37), macrophage infiltration (38), vascular smooth muscle cell proliferation (39), and collagen accumulation (40), ultimately resulting in aortic stiffening and vascular remodeling. Under conditions of sustained aldosterone elevation, excessive MR activation is considered to contribute to the development and progression of multisystem disorders, including arterial hypertension, heart failure, and diabetes-associated renal injury. Against this background, both steroidal and nonsteroidal MRAs confer protective effects on the heart, vasculature, and kidneys by blocking MR signaling and thereby attenuating inflammation, tissue remodeling, and fibrosis (29).

### 2.3 Residual cardiovascular risk suggests the involvement of non-MR pathways: growing attention to GPER-mediated rapid signaling

However, large clinical cohort studies indicate that even with MRA therapy, patients with PA continue to experience significantly higher rates of cardiovascular events than those with essential hypertension (hazard ratio ≈1.9), with an excess of approximately 14% in cardiovascular events over 10 years, and this difference appears to be independent of the level of blood pressure control (41). These findings suggest that the pathogenic effects of aldosterone are not entirely dependent on the canonical MR pathway but may also involve rapid signaling mediated by noncanonical receptors (**Figure 1**), such that conventional MR antagonism may be insufficient to fully block its biological actions. Recent studies have identified the G protein–coupled estrogen receptor (GPER1, also known as GPR30) as a mediator of aldosterone-induced rapid non-genomic signaling (42). It should be noted that rapid non-genomic actions of aldosterone are heterogeneous and include both MR-dependent and MR-independent mechanisms. While some rapid signaling events are mediated by membrane-associated MR, others may occur through MR-independent pathways such as GPER (43), which may partly explain why conventional MR antagonists cannot completely abolish certain rapid deleterious responses. This pathway is considered a key potential mechanism underlying the persistence of cardiovascular risk following MR blockade and has increasingly emerged as a focal point in research on aldosterone-related cardiovascular injury.

### 2.4 Immune–inflammatory mechanisms as central drivers of aldosterone-related cardiovascular injury

Evidence indicates that aldosterone activates immune responses and promotes the recruitment and polarization of inflammatory cells, with macrophages and T lymphocytes playing pivotal roles (44, 45). Aldosterone-induced MR activation drives macrophage infiltration and M1 polarization within cardiovascular tissues and upregulates multiple proinflammatory mediators, such as monocyte chemoattractant protein-1 (MCP-1) and tumor necrosis factor-α (TNF-α), thereby exacerbating local inflammatory responses (46–48). In addition, T cells within cardiovascular-associated immune populations also express functional MR and can be directly regulated by aldosterone (48, 49). Recent studies further demonstrate that human T cells co-express MR and GPER, suggesting that, in addition to its canonical genomic effects, aldosterone may also regulate T-cell activation and amplify inflammatory responses through GPER-mediated non-genomic pathways (49). Inflammation represents one of the most critical pathological features of aldosterone-induced vascular injury and exacerbates multiple vascular abnormalities, including vascular remodeling, endothelial dysfunction, and fibrosis (35). Vascular inflammation is closely associated with the incidence of cardiovascular disease and stroke (50, 51). From a mechanistic perspective, effective control of inflammation represents a key strategy for reducing the risk of cardiovascular complications (52), and may help prevent aldosterone-induced cardiac fibrosis and remodeling (53).

## 3 Immune–inflammatory mechanisms mediated by aldosterone

A substantial body of evidence indicates that aldosterone, through activation of the MR and the GPER, induces the expression of inflammatory mediators, upregulates adhesion molecules, and promotes immune cell infiltration, thereby triggering immune–inflammatory responses and playing a key role in the pathophysiological processes that drive cardiovascular injury (35). Under conditions of elevated aldosterone levels, effector cells such as macrophages, monocytes, and T lymphocytes become markedly activated, thereby amplifying the local inflammatory microenvironment and accelerating cardiovascular structural remodeling and functional deterioration.

### 3.1 Macrophage polarization and the activation of innate–adaptive immune crosstalk

In PA-related inflammation, aldosterone modulates the activation state of innate immune cells—primarily macrophages—and subsequently shapes adaptive immune responses dominated by T lymphocytes, thereby playing a pivotal role in the amplification of inflammation and the progression of tissue injury.

**Figure 2:**
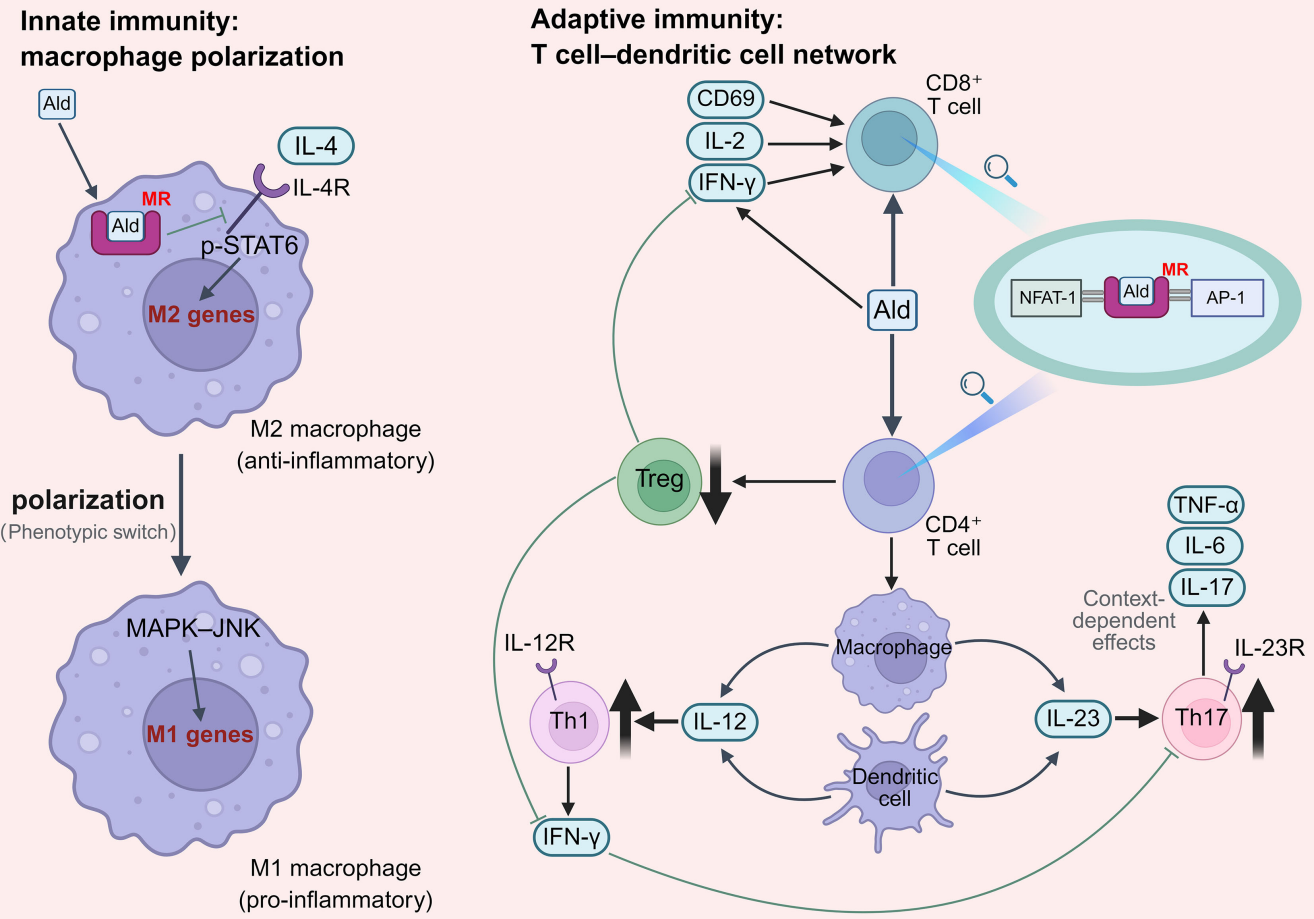
Aldosterone-driven innate–adaptive immune crosstalk through macrophage polarization and T cell–dendritic cell networks. This schematic illustrates how aldosterone shapes immune–inflammatory responses through mineralocorticoid receptor (MR)–dependent signaling across innate and adaptive immunity. In innate pathways, aldosterone–MR activation disrupts interleukin-4 receptor (IL-4R)–STAT6 signaling and activates the mitogen-activated protein kinase–c-Jun N-terminal kinase (MAPK–JNK) pathway, driving macrophage polarization from an anti-inflammatory M2 phenotype toward a proinflammatory M1 phenotype. In adaptive immunity, MR interaction with nuclear factor of activated T cells 1 (NFAT-1) and activator protein-1 (AP-1) enhances CD4^+^ T-cell differentiation toward Th1 and Th17 subsets while suppressing regulatory T cells (Tregs), and promotes CD8^+^ T-cell activation via upregulation of CD69, interleukin-2 (IL-2), and interferon-γ (IFN-γ). Dendritic cell– and macrophage-derived interleukin-12 (IL-12) and interleukin-23 (IL-23) support Th1 and Th17 differentiation, with the IL-17/IL-23 axis depicted as context-dependent in cardiovascular remodeling. Ald, aldosterone; MR, mineralocorticoid receptor; IL-4R, interleukin-4 receptor; MAPK, mitogen-activated protein kinase; JNK, c-Jun N-terminal kinase; NFAT-1, nuclear factor of activated T cells 1; AP-1, activator protein-1; Treg, regulatory T cell; IFN-γ, interferon-γ; IL-2, interleukin-2; IL-12, interleukin-12; IL-23, interleukin-23; Th1, T helper 1 cell; Th17, T helper 17 cell.

At the level of innate immunity, aldosterone regulates macrophage phenotypes through the MR (**Figure 2**). The interleukin-4 receptor (IL-4R) is a key signaling pathway driving the induction of the M2 macrophage phenotype. MR activation can interfere with IL-4 signaling (54) while activating the mitogen-activated protein kinase–c-Jun N-terminal kinase (MAPK–JNK) pathway (55), driving macrophage polarization from a reparative M2 phenotype toward a proinflammatory M1 phenotype and establishing a foundation for the sustained amplification of local inflammatory responses.

Beyond its direct effects on innate immune cells, aldosterone can also regulate adaptive immune responses through dendritic cell–dependent mechanisms (**Figure 2**). By activating MR, aldosterone promotes its interaction with nuclear factor of activated T cells 1 (NFAT-1) and activator protein-1 (AP-1) (56), thereby enhancing T-cell proliferation and tissue infiltration, promoting the differentiation of CD4^+^ T cells toward proinflammatory helper T-cell phenotypes Th1 and Th17, while concurrently suppressing the regulatory T-cell (Treg) population (56). A reduction in Tregs diminishes their suppressive control over interferon-γ (IFN-γ) production, thereby exacerbating target organ injury (56). Concurrently, aldosterone stimulation upregulates the expression of CD69, interleukin-2 (IL-2), and interferon-γ (IFN-γ), thereby enhancing CD8^+^ T-cell activation (57). MR also exerts regulatory effects on peripheral T cells, promoting tissue infiltration and enhancing the activation of double-positive CD4^+^ and single-positive CD8^+^ T-cell subsets (58). Collectively, these converging pathways drive the production of inflammatory mediators and ultimately lead to tissue injury.

It should be noted that the role of the interleukin-17/interleukin-23 (IL-17/IL-23) axis in aldosterone-related diseases is highly context-dependent. Interleukin-17 (IL-17), produced by Th17 cells, is a proinflammatory cytokine that is secreted by both innate and adaptive immune cells (59). The expansion and survival of Th17 cells depend on interleukin-23 (IL-23), which is secreted by activated dendritic cells and macrophages (60). However, in a deoxycorticosterone acetate (DOCA) combined with angiotensin II (Ang II)–induced high-aldosterone/high-salt model, genetic deficiency of IL-17 or IL-23 paradoxically accelerates renal injury, suggesting that the IL-17/IL-23 axis may contribute to the maintenance of immune homeostasis rather than acting solely as a proinflammatory pathway in specific pathological contexts (61). One possible explanation is that IL-17/IL-23 signaling may contribute to immune regulation by maintaining Th17 cell homeostasis and modulating interactions between innate and adaptive immune cells, thereby limiting excessive inflammatory damage in certain contexts. Accordingly, the canonical proinflammatory effects of MR activation and the context-specific protective roles of the IL-17/IL-23 axis should be interpreted in an integrated manner that accounts for disease state.

**Figure 3:**
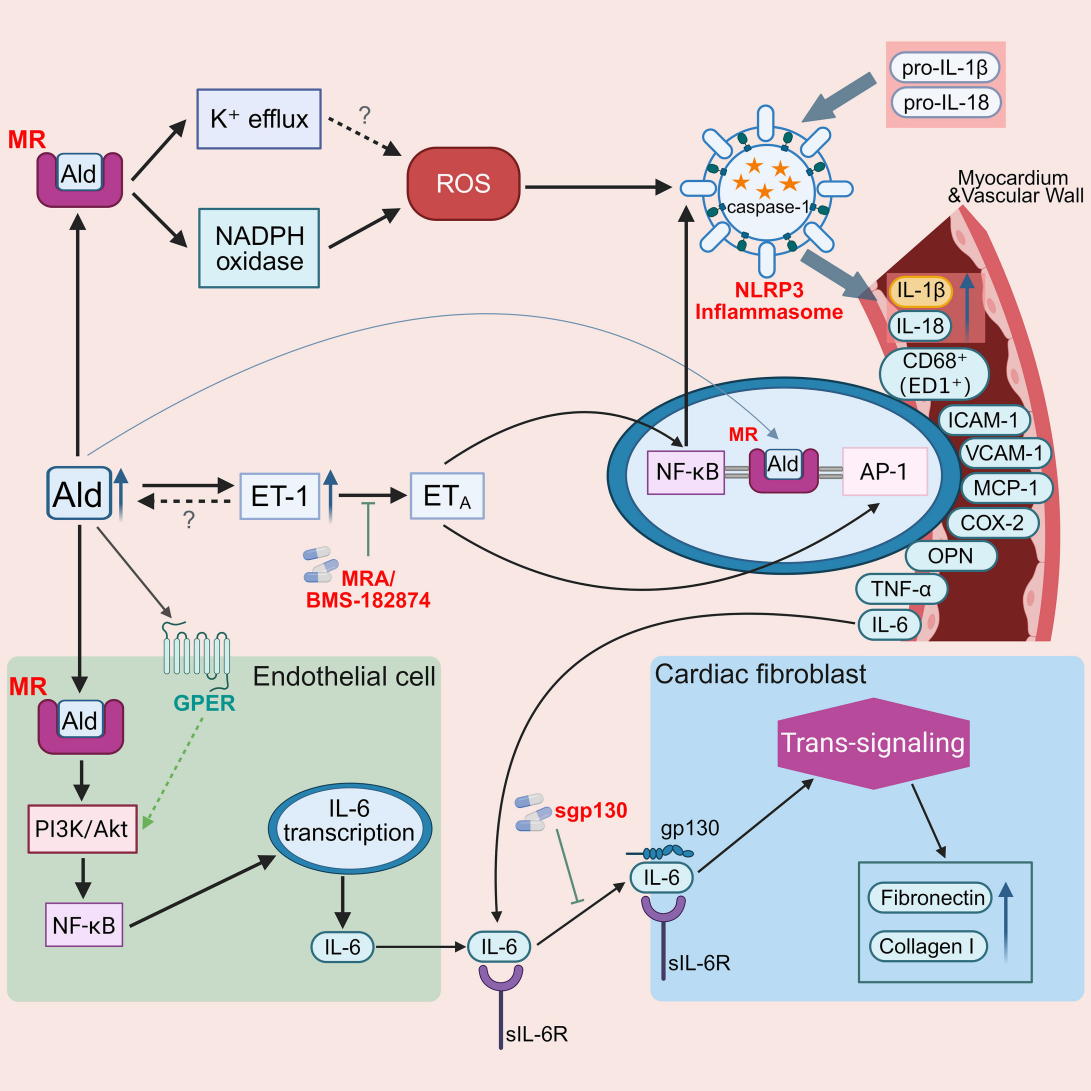
Inflammatory hubs downstream of aldosterone: NLRP3 inflammasome activation, adhesion molecules, ET-1/ET_A_ signaling, and IL-6 trans-signaling in cardiovascular remodeling. This schematic depicts how aldosterone–mineralocorticoid receptor (MR) signaling promotes vascular and myocardial injury through interconnected inflammatory pathways. Aldosterone induces reactive oxygen species (ROS) generation via NADPH oxidase and activates nuclear factor κB (NF-κB), driving NLR family pyrin domain-containing 3 (NLRP3) inflammasome assembly, caspase-1 activation, and the maturation of interleukin-1β (IL-1β) and interleukin-18 (IL-18) in macrophages. In parallel, aldosterone can enhance endothelin-1 (ET-1) production, and activation of the endothelin receptor type A (ET_A_) receptor further amplifies downstream inflammatory signaling pathways, contributing to NF-κB and activator protein-1 (AP-1) activation. NF-κB and activator protein-1 (AP-1) signaling upregulate endothelial adhesion molecules (ICAM-1, VCAM-1) and chemokines (MCP-1/CCL2), facilitating leukocyte recruitment and tissue infiltration. In endothelial cells, aldosterone stimulates interleukin-6 (IL-6) production via the phosphoinositide 3-kinase (PI3K)/NF-κB pathway. In addition to classical MR signaling, aldosterone may also activate the G protein–coupled estrogen receptor (GPER), which can engage PI3K/Akt-dependent pathways and potentially interact with MR-mediated inflammatory signaling. IL-6 trans-signaling through the soluble IL-6 receptor (sIL-6R) and gp130 in cardiac fibroblasts drives fibronectin and collagen I synthesis, thereby promoting myocardial fibrosis and cardiovascular remodeling. Ald, aldosterone; MR, mineralocorticoid receptor; ROS, reactive oxygen species; NLRP3, NLR family pyrin domain-containing 3; ICAM-1, intercellular adhesion molecule-1; VCAM-1, vascular cell adhesion molecule-1; MCP-1/CCL2, monocyte chemoattractant protein-1; ET-1, endothelin-1; ET_A_, endothelin receptor type A; PI3K, phosphoinosi-tide 3-kinase; NF-κB, nuclear factor κB; AP-1, activator protein-1; sIL-6R, soluble IL-6 receptor; gp130, glycoprotein 130.

Studies by Bruder-Nascimento et al. demonstrate that activation of the interleukin-1 receptor (IL-1R) plays an important role in aldosterone-induced vascular injury (62). NLRP3 is one of the most extensively studied members of the nucleotide-binding oligomerization domain (NOD)-like receptor (NLR) family and serves as a key innate immune sensor of tissue injury (63, 64), with its primary function being the regulation of inflammasome assembly. *In vitro* studies indicate that aldosterone induces the generation of reactive oxygen species (ROS) and activates nuclear factor κB (NF-κB) signaling, thereby promoting the release of NLRP3-dependent interleukin-1β (IL-1β) from bone marrow–derived macrophages, while concomitantly upregulating the expression of NLRP3, activated caspase-1, and mature IL-1β in human peripheral blood mononuclear cells (62) (**Figure 3**). Animal studies further demonstrate that in mice lacking interleukin-1 receptor (IL-1R) or the key inflammasome components NLRP3 and caspase-1, aldosterone-induced vascular dysfunction, inflammation, and remodeling are markedly attenuated (62).

Collectively, existing *in vitro* and *in vivo* evidence indicates that the NLRP3 inflammasome occupies a key regulatory position in aldosterone-mediated vascular inflammation and remodeling. Population-based studies further suggest that a high-aldosterone state is closely associated with systemic inflammatory activation and inflammation of the vascular wall (65). However, the direct role of the NLRP3–IL-1 axis in PA-related cardiovascular injury in humans remains to be validated by further clinical studies, and this pathway represents a promising potential target for anti-inflammatory therapeutic strategies in high-aldosterone states.

### 3.2 Upregulation of adhesion molecules and proinflammatory mediators

Concurrently, aldosterone markedly upregulates the expression of multiple leukocyte adhesion molecules and proinflammatory mediators, thereby promoting the recruitment and infiltration of inflammatory cells into cardiovascular tissues. In aldosterone/high-salt animal models, sustained aldosterone infusion over 1–4 weeks significantly increases tissue expression of intercellular adhesion molecule-1 (ICAM-1), vascular cell adhesion molecule-1 (VCAM-1), monocyte chemoattractant protein-1 (MCP-1/CCL2), as well as cyclooxygenase-2 (COX-2) and osteopontin (OPN) (66), accompanied by the accumulation of inflammatory cells—predominantly CD68^+^ (ED1^+^) monocytes/macrophages—around the myocardium and vascular wall (66). Together, these molecules synergistically promote immune cell adhesion, migration, and tissue infiltration, ultimately leading to pronounced myocardial and vascular inflammatory responses (**Figure 3**). Further studies reveal that aldosterone enhances the transcriptional activity of intercellular adhesion molecule-1 (ICAM-1) and connective tissue growth factor (CTGF) by activating serum/glucocorticoid-regulated kinase 1 (SGK1) and nuclear factor κB (NF-κB) signaling pathways, effects that may contribute to the progression of aldosterone-induced inflammation (67).

Notably, ICAM-1– and VCAM-1–mediated inflammatory cell adhesion is not only a hallmark of the inflammatory response but also represents a potential target for therapeutic investigation. In angiotensin II (Ang II)–based models, administration of an ICAM-1–neutralizing antibody dose-dependently lowers blood pressure, improves endothelium-dependent vasodilation, and significantly attenuates vascular wall thickening, myocardial hypertrophy, and fibrosis, while suppressing LFA-1^+^/CD68^+^ macrophage infiltration as well as the expression of proinflammatory cytokines such as interleukin-1β (IL-1β) and interleukin-6 (IL-6) and the activation of NF-κB (68, 69). Consistently, blockade of VCAM-1 also significantly reduces blood pressure in Ang II–induced mice, improves endothelial function, and concomitantly suppresses macrophage infiltration, oxidative stress, and vascular remodeling (70), thereby functionally and structurally substantiating the pathogenic role of adhesion molecules in RAAS-related vascular inflammation. However, the available interventional evidence is derived predominantly from animal models, and its translational relevance to conditions of isolated aldosterone excess or to PA remains to be established through further studies.

### 3.3 ET-1/ET_A_ signaling axis–mediated inflammatory injury

Endothelin-1 (ET-1) plays a critical role in the development and progression of multiple cardiovascular diseases (71). As an important proinflammatory regulator, ET-1 can induce the release of multiple inflammatory cytokines, including interleukin-1β (IL-1β), interleukin-6 (IL-6), and tumor necrosis factor-α (TNF-α) (72). PA is the most common endocrine cause of resistant hypertension (73), and affected patients exhibit an increased risk of cardiovascular and renal complications, accompanied by elevated circulating levels of ET-1 (71). In salt-loaded, aldosterone-infused animal models, aldosterone enhances local ET-1 signaling through an MR-dependent mechanism, which in turn activates downstream inflammatory transcriptional pathways via the endothelin type A receptor (ET_A_), markedly increasing AP-1 DNA-binding activity and amplifying NF-κB–related inflammatory signaling, thereby inducing vascular inflammatory responses accompanied by ED1^+^ macrophage infiltration (74). These changes can be markedly attenuated by either the ET_A_ receptor antagonist BMS-182874 or MR antagonists, with concomitant reductions in transcription factor activity and inflammatory responses (74). These findings suggest that aldosterone can amplify AP-1/NF-κB–dependent inflammatory transcriptional responses through the ET-1/ET_A_ signaling axis, potentially playing an important pathological role in aldosterone-related cardiovascular inflammatory injury and structural remodeling (74) (**Figure 3**).

Notably, a bidirectional regulatory relationship may exist between ET-1 and aldosterone. In endothelial-specific human ET-1–overexpressing mice, prolonged exposure (3 months) significantly elevates plasma aldosterone levels, whereas corticosterone levels remain unchanged. Treatment with an ET_A_ receptor antagonist partially reverses this effect, suggesting that endothelium-derived ET-1 may participate in the regulation of aldosterone secretion via ET_A_ receptor signaling (75). In addition, *EDN1* (encoding ET-1) expression can be detected in circulating extracellular vesicles (EVs) from patients with PA but not in those with essential hypertension. Following unilateral adrenalectomy, *EDN1* expression in EVs from patients with PA is significantly reduced, suggesting its potential utility as a biomarker for identifying PA and assessing disease status (76).

### 3.4 The role of interleukin-6 in cardiovascular remodeling

Low-grade chronic inflammation plays an important role in the process of aldosterone-induced cardiac fibrosis (77). Interleukin-6 (IL-6) is a representative proinflammatory cytokine closely associated with fibrosis, and previous studies indicate that its trans-signaling participates in the process of aldosterone-mediated myocardial fibrosis (53). Epidemiological evidence consistently supports IL-6 as a central mediator of cardiovascular risk, with elevated levels associated with an increased risk of cardiovascular mortality, major adverse cardiovascular events, myocardial infarction, stroke, peripheral arterial disease, and heart failure (78). Clinical studies show that plasma IL-6 levels are significantly higher in patients with PA than in those with essential hypertension, and are accompanied by a higher left ventricular mass index (LVMI), more severe myocardial fibrosis, and impaired diastolic function. IL-6 levels are positively correlated with 24-hour urinary aldosterone excretion and multiple echocardiographic parameters. Follow-up studies further demonstrate that IL-6 levels markedly decrease after unilateral adrenalectomy in patients with PA, accompanied by improvements in cardiac structure and diastolic function, supporting a close association between IL-6 and aldosterone-related myocardial remodeling (53).

At the mechanistic level, cell-based studies indicate that aldosterone induces endothelial cell expression and secretion of IL-6 through the MR/phosphoinositide 3-kinase (PI3K)/NF-κB signaling pathway. Subsequently, IL-6, via soluble IL-6 receptor α (sIL-6R)–mediated trans-signaling, acts on cardiac fibroblasts that express gp130 but lack membrane-bound IL-6Rα, thereby inducing the expression of collagen and multiple fibrosis-related factors (**Figure 3**). In aldosterone-infused mouse models, selective inhibition of IL-6 trans-signaling—such as by increasing or supplementing soluble gp130 or via sgp130 knock-in—attenuates cardiac hypertrophy and fibrosis, supporting a key mediating role for IL-6 trans-signaling in aldosterone-induced myocardial remodeling (53).

Given that IL-6 occupies an upstream hub in inflammatory responses and shows a relatively well-established causal association with cardiovascular events, it is considered one of the most promising targets for anti-inflammatory therapy (78). Several large cardiovascular outcomes trials (e.g., ZEUS and ARTEMIS) are currently evaluating whether anti–IL-6 monoclonal antibodies can further reduce residual risk on top of standard therapy (78). However, the measurement of IL-6 levels is subject to substantial variability and lacks standardized thresholds, and the long-term cardiovascular benefits and safety of sustained IL-6 signaling inhibition remain to be further validated. In PA, IL-6 represents a critical node in MR-driven chronic inflammation, myocardial remodeling, and vascular injury, and may serve as a future tool for risk stratification as well as a potential combinatorial target alongside MRAs or ASIs. Notably, strategies that selectively block IL-6 trans-signaling (e.g., sgp130) have demonstrated pronounced anti–myocardial fibrotic effects in animal models, suggesting a potential novel therapeutic avenue beyond the MR axis.

### 3.5 Animal and clinical evidence supporting a central role for inflammation in PA-related cardiovascular injury

Evidence from both animal models and clinical studies collectively supports a critical role for inflammation in PA-related cardiovascular injury. In the context of aldosterone/MR overactivation, immune cells such as monocytes/macrophages and T cells adopt a proinflammatory phenotype, driving the formation of local inflammatory microenvironments in the vasculature and myocardium and closely associating with vascular remodeling, tissue fibrosis, and hypertensive phenotypes (48). In population-based studies, imaging evidence (e.g., positron emission tomography [PET]) further demonstrates that arterial wall inflammation is significantly higher in patients with PA than in those with essential hypertension (65). The convergence of these animal and human studies establishes a central role for immune inflammation in the initiation and progression of PA-related cardiovascular injury.

Notably, mineralocorticoid receptor antagonism can significantly attenuate aldosterone-mediated inflammatory responses. Following MR antagonist therapy, levels of inflammatory biomarkers decrease and inflammatory cell infiltration in the myocardium and vasculature is reduced, suggesting that blockade of the aldosterone/MR axis can partially reverse inflammation-mediated cardiovascular injury and thereby supporting the potential of inflammatory pathways as therapeutic targets for cardiovascular protection in PA (79). For example, MR antagonists reduce inflammatory cell infiltration (monocytes/macrophages) and associated inflammatory biomarkers linked to focal ischemic and necrotic changes, as well as to vascular, myocardial, and renal injury (66, 74). These findings suggest that blockade of the aldosterone/MR axis not only exerts antihypertensive effects but also partially reverses PA-related cardiovascular injury by suppressing inflammatory pathways, thereby providing an important theoretical basis for MR-targeted anti-inflammatory and organ-protective strategies.

## 4 Novel mineralocorticoid receptor antagonists

### 4.1 Limitations of conventional MRAs

MRAs are broadly classified into steroidal and nonsteroidal categories. Spironolactone and eplerenone, as conventional steroidal MRAs, differ substantially from newer nonsteroidal MRAs in their physicochemical properties, receptor selectivity, and modes of receptor binding (80). Animal studies indicate that steroidal MRAs exhibit pronounced tissue distribution bias *in vivo*. The concentrations of spironolactone and its active metabolites in the kidney can reach approximately sixfold those in the heart, whereas for eplerenone the ratio is approximately threefold (81). This distribution pattern confers strong efficacy in inhibiting renal sodium reabsorption and controlling blood pressure, but achieving sufficient pharmacologically-active concentrations in nonrenal tissues such as the heart often requires higher doses. In addition, the receptor-binding conformations of steroidal MRAs and their capacity to recruit coactivators exhibit tissue-specific differences, which further constrain their protective effects in cardiovascular tissues (81).

Spironolactone and eplerenone have been clinically proven to lower blood pressure and improve outcomes in heart failure; however, both agents are associated with notable limitations. Although spironolactone exhibits potent MR antagonistic efficacy, its limited receptor selectivity leads to off-target binding to androgen and progesterone receptors, resulting in hormone-related adverse effects such as gynecomastia, sexual dysfunction, and menstrual irregularities (80, 81). By contrast, eplerenone exhibits higher MR selectivity; however, its antagonistic potency is only approximately one-fortieth that of spironolactone (81), and achieving sufficient antagonistic effects in target tissues such as the heart typically requires higher doses. Although hormone-related adverse effects are reduced, the risk of hyperkalemia is not completely eliminated (80, 81). Notably, eplerenone often requires dosing levels far exceeding its natriuretic effective dose—approximately 30–100-fold higher—to achieve significant cardioprotective effects (82), highlighting a dose-dependent limitation of conventional steroidal MRAs in cardiovascular protection.

Collectively, these features indicate that although steroidal MRAs remain a cornerstone of pharmacological therapy for PA, unmet clinical needs persist in achieving adequate cardiovascular protection and long-term safety. To systematically compare the differences between conventional steroidal and newer nonsteroidal MRAs in terms of pharmacological properties, tissue distribution, receptor selectivity, safety profiles, and PA-related clinical evidence, these characteristics are summarized in **Table 1**.

**Table 1:** Comparative pharmacological and clinical characteristics of steroidal and nonsteroidal mineralocorticoid receptor antagonists.

Characteristics	Spironolactone	Eplerenone	Finerenone	Esaxerenone
Type	Steroidal	Steroidal	Nonsteroidal	Nonsteroidal
Tissue distribution	Kidney > Heart	Kidney > Heart	Balanced (Heart/Kidney)	Balanced (Heart/Kidney)
Half-life (t½)	Up to 16.5 h (including active metabolites)	4–6 h	2–3 h	17–20 h
MR selectivity	Low (significant AR/PR cross-reactivity)	Moderate (minimal AR/PR activity)	High (>500-fold selectivity)	Very high (>1000-fold selectivity)
MR inhibitory potency (IC50)	66 nM	970 nM	18 nM	3.7 nM (lowest)
Sex hormone–related adverse effects	Common (gynecomastia, sexual dysfunction, menstrual irregularities)	Less frequent	None reported	None reported
Risk of hyperkalemia	High	Moderate	Moderate (generally manageable)	Moderate (overall favorable)
Blood pressure–lowering effect	Strong (10–20+ mmHg)	Moderate	Mild (2–4 mmHg)	Strong (10–20 mmHg)
Cardiovascular outcome evidence	Substantial (RALES, PATHWAY)	Limited	Strong (FIDELIO-DKD, FIGARO-DKD)	Robust preclinical evidence; early clinical data
Efficacy in PA	Standard therapy	Second-line; less effective than spironolactone	Exploratory use; limited evidence, not sufficient to replace steroidal MRAs, with signals of potential limitations	Relatively robust evidence (Japanese prospective and real-world studies), suggesting potential value as a first-line alternative in selected patient populations

IC50 values are derived from *in vitro* assays and may vary across experimental systems. Cardiovascular outcome evidence is primarily based on large trials in heart failure and/or chronic kidney disease, and PA-specific data for nonsteroidal MRAs remain limited.

MR, mineralocorticoid receptor; MRA, mineralocorticoid receptor antagonist; PA, primary aldosteronism; BP, blood pressure; AR, androgen receptor; PR, progesterone receptor; IC50, half-maximal inhibitory concentration. This table compares tissue distribution, pharmacokinetics, mineralocorticoid receptor (MR) selectivity and inhibitory potency (IC50), key adverse effects, blood pressure–lowering effects, cardiovascular outcome evidence, and available data in primary aldosteronism (PA) for representative MRAs (spironolactone, eplerenone, finerenone, and esaxerenone).

### 4.2 Novel nonsteroidal MRAs: pharmacological advantages and mechanistic advances

#### 4.2.1 Finerenone: cardiorenal selectivity and evidence in primary aldosteronism

Finerenone is a novel, high-affinity nonsteroidal mineralocorticoid receptor antagonist (MRA) with more than 500-fold selectivity for the MR and minimal activity toward other steroid receptors or ion channels. Unlike steroidal MRAs, finerenone has a relatively short plasma half-life (approximately 2–3 hours) and no active metabolites, and exhibits a more balanced distribution between the heart and kidney rather than a renal bias (**Table 1**), a property that is thought to enhance organ-protective effects while maintaining electrolyte homeostasis (82, 83).

In the DOCA/salt-loaded model, finerenone markedly attenuates cardiac hypertrophy, reduces brain natriuretic peptide (BNP) levels and proteinuria, and is superior to eplerenone in suppressing myocardial fibrosis as well as glomerular and tubular injury. In a post–myocardial infarction chronic heart failure model, even low-dose finerenone (1 mg/kg) improves left ventricular systolic and diastolic function and reduces BNP as well as inflammatory and fibrotic markers, whereas eplerenone (100 mg/kg) fails to confer comparable protective effects in the same model. Moreover, in animal studies, finerenone does not induce overt hyperkalemia, suggesting that it exerts cardioprotective effects with relatively minimal disruption of renal electrolyte homeostasis (82).

In clinical studies, finerenone has demonstrated the potential to reduce the risk of cardiovascular and renal events associated with chronic kidney disease related to type 2 diabetes mellitus (T2DM-CKD) and has been approved for use in this population (84, 85). In the phase III FIDELIO-DKD and FIGARO-DKD trials, finerenone exhibited relatively modest blood pressure–lowering effects (on average, approximately 2–4 mmHg) yet significantly reduced composite cardiovascular endpoints, with particularly consistent and robust benefits in reducing hospitalizations for heart failure, suggesting that its cardioprotective effects are not primarily driven by blood pressure reduction but are more likely related to the suppression of MR-mediated inflammatory and fibrotic processes (86, 87).

The clinical utility of finerenone in PA remains exploratory, and the available evidence is currently insufficient to supplant steroidal MRAs (85). An exploratory randomized controlled trial demonstrated that finerenone can achieve short-term blood pressure reduction in patients with PA, with antihypertensive efficacy comparable to spironolactone, but with milder increases in serum potassium and no observed sex hormone–related adverse effects, suggesting potential advantages in safety and tolerability (88). However, its long-term antihypertensive efficacy and cardiovascular protective effects remain to be validated in larger cohorts with extended follow-up.

Notably, a recent real-world study in patients with PA applying a conversion scheme of “50 mg eplerenone ≈ 10 mg finerenone” found a lower rate of complete clinical and biochemical remission during finerenone treatment, accompanied by persistent renin suppression, which is generally associated with a higher cardiovascular risk (89). Notably, the pharmacokinetic characteristic of finerenone, particularly its relatively short plasma half-life, may partly explain the relatively modest biochemical remission rates observed in some real-world studies of PA treatment. These findings suggest that therapeutic responses to finerenone in PA may be influenced by factors such as dosing, aldosterone burden, patterns of MR activation, and tissue distribution characteristics, and that there may be signals of insufficient efficacy in achieving biochemical treatment targets in PA. Given that this was a noninterventional observational study with a limited sample size, the clinical significance of these observations requires clarification through larger, prospective randomized controlled trials (89). Although the magnitude of potassium elevation with finerenone appears relatively modest in PA studies, the hyperkalemia risk observed in CKD and heart failure populations underscores the need for enhanced electrolyte monitoring when finerenone is used in patients with PA (90). Whether finerenone confers cardiovascular protective effects in PA that are distinct from those of steroidal MRAs remains to be determined through further investigation.

#### 4.2.2 Esaxerenone: emerging evidence in salt-sensitive hypertension and primary aldosteronism

Esaxerenone is a recently developed nonsteroidal mineralocorticoid receptor antagonist. *In vitro* studies indicate that, compared with spironolactone and eplerenone, esaxerenone exhibits greater potency and selectivity for the MR (**Table 1**), with more than 1,000-fold MR selectivity and MR-binding affinity approximately fourfold and seventy-sixfold higher than that of spironolactone and eplerenone, respectively (91). In addition, esaxerenone exhibits favorable pharmacokinetic properties, including a relatively long half-life that allows once-daily dosing and contributes to sustained antihypertensive efficacy in clinical studies (92). Structural studies show that esaxerenone forms a specific binding conformation with the MR ligand-binding domain (MR-LBD) and, through its relatively bulky side chain, achieves stable interactions that underlie its high affinity and marked selectivity (91). Phase III clinical trial results indicate that esaxerenone is effective and well tolerated in patients with hypertension, and it has been in clinical use for this indication in Japan since May 2019 (84, 85, 93).

In animal models, esaxerenone exerts significant protective effects against cardiac injury induced by high-salt loading and aberrant MR activation (94). In the Dahl salt-sensitive rat model, esaxerenone reduces systolic blood pressure by approximately 20 mmHg and improves long-term survival; decreases interstitial and perivascular collagen deposition in the myocardium; downregulates fibrosis-related genes, including transforming growth factor-β (TGF-β), type I and III collagens, and plasminogen activator inhibitor-1 (PAI-1); suppresses the aberrant upregulation of serum/glucocorticoid-regulated kinase 1 (SGK1) downstream of aldosterone/angiotensin II (Ang II), thereby blocking SGK1-mediated pathways of myocardial fibrosis and hypertrophy; and concurrently lowers the expression of inflammatory cytokines such as tumor necrosis factor-α (TNF-α) and interleukin-6 (IL-6), while attenuating excessive reactive oxygen species (ROS) generation (94).

Beyond the heart, esaxerenone also exhibits important protective effects in the kidney and across inflammation-related mechanisms (95). In a rat model of sustained aldosterone infusion combined with a high-salt diet, esaxerenone suppresses MR activation and nuclear translocation, significantly reduces CD8^+^ T-cell infiltration in renal tissue, and downregulates both the expression and phosphorylation of the distal convoluted tubule Na^+^–Cl^-^ cotransporter (NCC). By concurrently inhibiting immune cell infiltration and ion transport activation, esaxerenone attenuates the progression of salt-sensitive hypertension and associated renal injury at multiple mechanistic levels (95). Overall, studies in animal models indicate that esaxerenone is highly effective in suppressing myocardial fibrosis, hypertrophy, and inflammatory oxidative stress, thereby validating the critical role of MR-mediated cardiorenal injury and highlighting the potential of MR antagonism in preventing PA-related cardiac and renal complications.

In clinical studies, a multicenter open-label trial conducted by Satoh et al. systematically evaluated the efficacy and safety of esaxerenone in patients with PA and concomitant hypertension (96). After 12 weeks of esaxerenone treatment in 44 rigorously diagnosed patients with PA, seated systolic/diastolic blood pressure decreased by 17.7/9.5 mmHg from baseline (both P < 0.0001), and 47.7% of patients achieved target blood pressure <140/90 mmHg, with antihypertensive effects evident as early as week 2 and sustained thereafter. Concomitant increases in plasma aldosterone concentration (PAC) and plasma renin activity (PRA), along with a reduction in the aldosterone-to-renin ratio (ARR), indicate effective MR blockade and relief of RAAS suppression, consistent with biochemical remission. In terms of safety, only approximately 4.5% (2 patients) experienced mild to moderate hyperkalemia or declines in estimated glomerular filtration rate (eGFR), and no cases of gynecomastia or breast tenderness were observed, suggesting overall good tolerability. Collectively, this study provides the first clinical evidence supporting the use of esaxerenone in PA and, from both blood pressure control and RAAS remodeling perspectives, underscores its potential cardiorenal protective value (96).

Real-world studies further provide supportive evidence for the use of esaxerenone in PA. In a single-center retrospective cohort study conducted by Fujimoto et al. (97), 87 patients with PA were enrolled, approximately one-third of whom received esaxerenone as initial therapy, while the remainder were switched from prior MRAs. After approximately 2 months of follow-up, systolic blood pressure decreased significantly from baseline (P = 0.025), and diastolic blood pressure showed a downward trend (P = 0.096). When a reduction in systolic or diastolic blood pressure of ≥10 mmHg was defined as a “response,” responders were significantly older on average (61.9 vs. 50.7 years, P = 0.0035), suggesting that older patients with PA may be more sensitive to MR antagonism. This observation is consistent with the subgroup trend from the phase III ESAX-HTN trial indicating greater benefit in older populations (98), providing additional support for the precision application of esaxerenone in specific PA phenotypes. Biochemically, esaxerenone was associated with a mild increase in serum potassium (approximately 3.9 → 4.0 mmol/L) and a small decline in eGFR, neither of which necessitated dose adjustment or treatment discontinuation; urinary albumin-to-creatinine ratio (UACR) and BNP levels showed downward trends but did not reach statistical significance. Despite the relatively short follow-up period, these findings suggest that esaxerenone provides reliable blood pressure control in PA and may confer cardiorenal benefits through RAAS modulation and improvements in microalbuminuria (97). In a retrospective analysis by Naruke et al. of patients with resistant hypertension complicated by heart failure, esaxerenone treatment was associated with a reduction in BNP levels, suggesting a potential alleviation of ventricular wall stress or volume overload and providing further clinical support for the cardioprotective potential of esaxerenone (99).

### 4.3 Positioning of nonsteroidal MRAs in PA: complementary rather than substitutive

As summarized in **Table 1**, nonsteroidal MRAs possess structural advantages in receptor selectivity and safety profiles; however, evidence regarding their ability to achieve biochemical remission in PA and to improve long-term cardiovascular outcomes remains limited, suggesting that they are more likely to serve as complementary options rather than direct substitutes for conventional steroidal MRAs. Current evidence suggests that finerenone is more oriented toward potential cardiorenal protective effects, whereas esaxerenone has relatively stronger evidence for blood pressure control and RAAS remodeling. These differences indicate that future MR-targeted therapy in PA may need to be tailored to disease phenotypes and therapeutic goals, with the development of more precise, individualized treatment strategies.

## 5 GPER signaling pathway: non-genomic effects of aldosterone and emerging targeted therapies

A substantial body of evidence indicates that, beyond the classical MR-mediated genomic effects, aldosterone can also trigger a series of rapidly occurring non-genomic responses. These rapid actions include both MR-dependent and MR-independent mechanisms. As residual cardiovascular risk following MR antagonism has gained increasing attention, recent studies have suggested that GPER may mediate part of aldosterone’s rapid signaling, thereby providing a new perspective for understanding its non-genomic effects and the associated mechanisms of cardiovascular injury (100, 101).

### 5.1 Molecular characteristics of GPER1 and an overview of GPER-mediated non-genomic aldosterone signaling

GPER1 (also known as GPR30, G protein–coupled estrogen receptor 30) (49) was initially identified as a receptor for 17β-estradiol (102); more recent studies further indicate that aldosterone can also mediate rapid non-genomic effects through GPER1-dependent signaling pathways across multiple tissue contexts. In cardiomyocytes, aldosterone enhances Na^+^/HCO_3_^-^ cotransporter (NBC) activity through GPER1-mediated generation of reactive oxygen species and activation of the PI3K–AKT signaling pathway (103). At the level of central vagal regulation, aldosterone triggers inositol 1,4,5-trisphosphate (IP_3_) receptor–dependent Ca²^+^ release accompanied by P/Q-type Ca²^+^ influx via GPER1, thereby modulating neuronal excitability and heart rate (104). In the tumor microenvironment, although aldosterone does not directly bind GPER1, the receptor functions as a node for signal integration and amplification, coordinating MR and epidermal growth factor receptor (EGFR) signaling to promote cell proliferation and migration (105).

In a recombinant GPER1 system established in Sf9 cells, aldosterone exhibits numerically higher competitive binding affinity for GPER1 than estradiol (106). Based on binding and functional assays in this recombinant system, aldosterone may, under specific experimental conditions, directly interact with GPER1 and thereby partially mediate its GPER1-dependent biological effects (106). However, these findings are derived primarily from recombinant animal cell systems, and their physiological relevance remains debated, requiring further validation in models that more closely recapitulate *in vivo* conditions.

### 5.2 Context-dependent effects of GPER in the regulation of vascular tone

GPER signaling mediates estrogen-dependent rapid vasodilation and nitric oxide (NO) production, thereby reducing peripheral resistance and inhibiting vascular remodeling, and conferring important cardiovascular protective effects (107). Further studies indicate that GPER1 activation improves myocardial Ca²^+^ homeostasis, suppresses oxidative stress and adverse cardiac remodeling, attenuates cardiac hypertrophy, and ameliorates structural and functional abnormalities associated with heart failure, with particularly promising therapeutic potential observed in postmenopausal females and experimental models (108). However, under aldosterone stimulation, Gros et al. observed in isolated coronary artery models that aldosterone can enhance vasoconstrictive responses through GPER1 activation, involving multiple rapid non-genomic signaling pathways, including ERK phosphorylation, and further modulating vascular smooth muscle cell apoptosis and myosin light chain (MLC) phosphorylation, thereby influencing vascular tone and contractile responses (101, 109). In addition, evidence suggests an association between genetic variants in GPER1 and hypertension (110).

Collectively, these lines of evidence suggest that, within the GPER context, estrogen and aldosterone may engage distinct signaling programs and elicit divergent biological outcomes. Accordingly, in high-aldosterone states such as PA, GPER1-mediated non-genomic signaling may operate in parallel with the classical MR pathway and contribute to the regulation of vascular tone and cardiovascular injury independently of MR-dependent transcriptional control. These findings provide a hypothetical mechanistic basis for understanding residual cardiovascular risk after MR blockade in PA and suggest that GPER warrants investigation as a potential complementary therapeutic target.

### 5.3 GPER-mediated immuno-inflammatory regulation: divergent effects under estrogen and aldosterone paradigms

The role of GPER signaling in immuno-inflammatory regulation remains controversial, with its functional effects exhibiting marked dependence on both ligand type and disease context. In estrogen-based experimental models, a study by Jacenik et al. demonstrated GPER1-mediated anti-inflammatory effects in a murine model of Crohn’s disease (111). However, a study by Cai et al. showed that estrogen, acting through the membrane receptor GPER1, promotes systemic lupus erythematosus (SLE) IgG–induced monocyte activation and exacerbates SLE serum–mediated cutaneous inflammatory responses (112). Findings by Heublein et al. indicate that GPER1 expression is upregulated in the ovary in models of ovarian endometriosis and pelvic inflammatory disease, suggesting a role for GPER1 in promoting ovarian inflammation (113). By contrast, studies examining GPER-mediated inflammatory responses with aldosterone as the ligand remain relatively limited. In a human umbilical vein endothelial cell (HUVEC) model, Tang et al. suggested that GPER1 may participate in aldosterone-induced endothelial inflammatory responses, at least in part, through the PI3K signaling pathway (114). Under conditions of MR knockdown, the GPER1 antagonist G15 still significantly downregulated inflammatory markers, including IL-1β/NLRP3 and the adhesion molecules ICAM-1 and VCAM-1 (114). Mechanistically, inhibition of PI3K partially attenuated GPER1-dependent inflammatory signaling but did not fully abrogate it, suggesting the involvement of parallel pathways, such as MAPK, in addition to PI3K (114). Further animal experiments demonstrated that, under high-aldosterone conditions, GPER1 deficiency, on top of MR antagonism, significantly reduced diastolic blood pressure and attenuated vascular inflammatory responses, as evidenced by decreased levels of IL-1β, NLRP3, and CD68 (115). Collectively, these findings suggest that, even under conditions of MR pathway inhibition, aldosterone may sustain immuno-inflammatory activation through GPER-mediated non-genomic mechanisms, providing a plausible explanation for the persistence of residual cardiovascular risk in PA despite MR blockade.

### 5.4 Targeting GPER for the modulation of cardiac remodeling and its potential relevance in primary aldosteronism

In neonatal rat cardiomyocytes (NRCMs), aldosterone-induced hypertrophy is primarily mediated by MR; the GPER1-specific agonist G-1 completely blocks the aldosterone-induced increase in cardiomyocyte surface area and, even after hypertrophy has been established, promotes its reversal within 24 hours. Correspondingly, this protective effect is abolished by GPER1 antagonists (G-15/G-36) or GPER1 knockdown using siGPER (116). It should be noted that the biological effects of GPER1 exhibit marked context dependence: under pathological conditions such as high-aldosterone states, GPER1 may participate in inflammatory responses and signaling pathways associated with cardiovascular injury; whereas under controlled pharmacological activation (e.g., with G-1), GPER1 in cardiomyocytes displays anti-hypertrophic and anti-remodeling effects. This divergence suggests that GPER1 is not a unifunctional receptor but rather a regulatory node whose actions depend on ligand characteristics, cell type, and the state of the surrounding signaling network. Collectively, GPER-mediated non-genomic signaling plays an important role in myocardial hypertrophy, vascular remodeling, inflammation, and fibrosis, positioning GPER as a potential therapeutic target for intervention in PA-related cardiovascular injury.

Building on the mechanistic evidence outlined above, pharmacological targeting of GPER has demonstrated substantial potential in preclinical studies. The synthetic GPER1 agonist G-1 is a nonsteroidal compound with high selectivity and affinity that binds specifically to GPER1 without interacting with the classical estrogen receptors ERα and ERβ (117). G-1 markedly attenuates angiotensin II (Ang II)–induced cardiomyocyte hypertrophy and downregulates the mRNA expression levels of atrial natriuretic peptide (ANP) and B-type natriuretic peptide (BNP) (118). Activation of GPER1 is closely associated with reduced collagen deposition (119). In an ovariectomized mouse model, treatment with G-1 significantly decreased myocardial infarct size and attenuated myocardial fibrosis (119). Wang et al. confirmed the expression of GPER1 in adult rat cardiac fibroblasts. In an ovariectomized rat model, G-1 treatment reduced the number of left ventricular fibroblasts and Ki-67–positive cells, attenuating estrogen deficiency–associated cardiac fibrosis and suggesting that activation of GPER may play an important role in anti-fibrotic processes in the heart (120). In an *in vivo* model of spontaneously hypertensive rats (SHRs), continuous infusion of G-1 for 28 days significantly attenuated myocardial hypertrophy and reduced levels of heart failure–related markers, including ANP and BNP; echocardiographic assessment further demonstrated improvements in myocardial structural remodeling, and these effects were independent of changes in blood pressure (116). Overall, G-1–induced activation of GPER1 may exert protective effects against pathological cardiac remodeling by suppressing hypertrophic and fibrotic responses and reducing levels of remodeling-associated cardiac markers.

G15 and G36 have been identified as selective antagonists of GPER1 and are commonly used to validate GPER1-mediated effects (121). In an ovariectomized female diabetic rat model, G15 blocked the cardioprotective effects mediated by estradiol (E2) and G-1, indicating that these actions are dependent on GPER1 (122). In isolated male rat cardiomyocytes, G-36 blocked the negative inotropic effects mediated by estradiol (E2) or G-1 (123). Notably, the clinically used steroidal MR antagonists eplerenone and spironolactone have been shown to partially inhibit G-1–induced extracellular signal–regulated kinase (ERK) phosphorylation in GPER1-expressing cells, indicating that both agents act as partial antagonists of GPER1 (109), yet this lack of selectivity may be insufficient to fully block all deleterious aldosterone-driven signaling.

At present, GPER1-targeted agents remain at an early stage of development. In theory, GPER1 agonists may recapitulate the cardiovascular protective effects of estrogen, whereas GPER1 antagonists could selectively block aldosterone-mediated non-genomic deleterious signaling. In terms of potential indications, populations with high-aldosterone states such as PA may represent the most direct candidates for GPER-targeted therapeutic strategies, with the goal of further reducing residual cardiovascular risk on top of conventional MR-based treatment. Moreover, given the involvement of GPER signaling in the pathophysiology of hypertension, heart failure, and metabolic disorders, its targeted modulation may also be extended in the future to anti-inflammatory and anti-fibrotic strategies for cardiovascular protection.

## 6 Aldosterone synthase inhibitors: from enzyme selectivity to clinical translation

### 6.1 Limitations of conventional therapies and the rationale for aldosterone synthase inhibitors

Although MRAs remain the cornerstone of therapy for PA and resistant hypertension, their ability to fully suppress the biological actions of aldosterone is inherently incomplete. Steroidal MRAs are constrained by limited receptor selectivity and are therefore prone to sex hormone–related adverse effects, whereas nonsteroidal MRAs, despite improved selectivity and tolerability, retain an inherent risk of hyperkalemia, particularly in patients with impaired renal function. Moreover, MRA therapy can trigger compensatory activation of the renin–angiotensin–aldosterone system, leading to elevations in both renin and aldosterone levels (124). This compensatory response may not only attenuate the effectiveness of MR blockade, necessitating higher clinical doses of MRAs, but may also potentiate MR-independent actions of aldosterone. Given that aldosterone exerts both genomic and non-genomic effects—of which the latter are not fully suppressed by MR antagonism—the overall therapeutic benefit of MRA-based strategies remains intrinsically limited (125, 126).

Accordingly, by targeting *CYP11B2* and suppressing aldosterone biosynthesis at its source, ASIs may, in principle, attenuate both the genomic and non-genomic actions of aldosterone and indirectly modulate downstream pathways such as reactive oxygen species generation and NLRP3/IL-1β–mediated inflammatory signaling, thereby conferring potential organ-protective effects (127, 128). Aldosterone biosynthesis is initiated by steroidogenic acute regulatory protein (StAR)–mediated transport of cholesterol into the mitochondria, followed by stepwise enzymatic conversion; however, the rate-limiting determinant of aldosterone production is the zona glomerulosa–specific enzyme *CYP11B2*, which catalyzes the terminal 11β-hydroxylation, 18-hydroxylation, and 18-oxidation of deoxycorticosterone (DOC) (5, 129). This key cytochrome P450 enzyme not only confers adrenal specificity for aldosterone synthesis through its tissue-restricted expression, but also delineates the divergent biosynthetic pathways of aldosterone and cortisol by serving as a differentiation node from *CYP11B1*, which is predominantly expressed in the zona fasciculata; moreover, during chronic regulation, alterations in *CYP11B2* expression largely govern the overall capacity for aldosterone production (5). Accordingly, the adrenal capacity for aldosterone synthesis is determined to a large extent by the transcriptional regulation of *CYP11B2*. Aldosterone and cortisol biosynthesis are closely related, as this enzyme exhibits a high degree of homology with the 11β-hydroxylase (*CYP11B1*) that catalyzes cortisol synthesis in the zona fasciculata; notably, the *CYP11B1* and *CYP11B2* genes are arranged in tandem on human chromosome 8q21–22, separated by an approximately 40-kb nucleotide sequence (130). The amino acid sequence of human *CYP11B2* shares approximately 93% homology with that of the 11β-hydroxylase encoded by *CYP11B1* (131). This high degree of homology renders the selective inhibition of *CYP11B2* particularly challenging. To facilitate a systematic comparison of ASIs across different generations in terms of *CYP11B2* selectivity, pharmacological properties, safety profiles, and PA-related clinical evidence, these key features are summarized in **Table 2**.

**Table 2:** Pharmacological characteristics, selectivity profiles, and clinical evidence of first- and second-generation aldosterone synthase inhibitors.

Drug	Generation	*CYP11B2* selectivity	Mechanistic features	BP effect	Evidence in PA	Key limitations/safety
Osilodrostat (LCI699)	First	Low	Reversible competitive inhibition of aldosterone synthase; dose-dependent off-target *CYP11B1* inhibition	Variable; generally weaker than MRAs	Early small-scale studies show reductions in aldosterone levels and improvement in hypokalemia; however, limited selectivity and safety concerns restrict its application in PA, and it is currently mainly used in Cushing’s syndrome.	Cortisol suppression at higher doses; narrow therapeutic window; adrenal insufficiency risk
Baxdrostat	Second	High (*CYP11B2*/*CYP11B1* ≈ 100:1)	Highly selective *CYP11B2* inhibition; preserved cortisol synthesis	Strong BP reduction in resistant hypertension	Phase II (SPARK II, n=15): ↓ aldosterone, improved hypokalemia; PA-specific trials ongoing	Dose-dependent hyperkalemia; reversible eGFR decline at high dose
Lorundrostat	Second	High (*CYP11B2*/*CYP11B1* ≈374:1)	Optimized *CYP11B2* selectivity; preserved cortisol synthesis	Strong BP reduction in uncontrolled and resistant hypertension	Emerging; PA-specific data limited	Electrolyte abnormalities (hyperkalemia, hyponatremia)

*CYP11B2* selectivity reflects *in vitro* inhibition ratios versus *CYP11B1* and may vary across assay systems. Evidence in PA is mainly derived from early-phase studies or ongoing PA-specific trials.

ASI, aldosterone synthase inhibitor; PA, primary aldosteronism; BP, blood pressure; eGFR, estimated glomerular filtration rate; MRA, mineralocorticoid receptor antagonist. This table summarizes *CYP11B2* selectivity, mechanistic features, blood pressure–lowering effects, available evidence in primary aldosteronism (PA), and key safety considerations for representative ASIs (osilodrostat, baxdrostat, and lorundrostat).

### 6.2 Osilodrostat (LCI699): first-generation aldosterone synthase inhibitor

Osilodrostat (LCI699) was derived through structural optimization of the lead compound FAD286 and represents the first orally available, reversible, competitive inhibitor of human aldosterone synthase (132). Across multiple phase I/II clinical studies, LCI699 markedly suppressed aldosterone production, achieving reductions of approximately 70–80% in circulating aldosterone levels and, to some extent, ameliorating hypokalemia (132–137).

Notably, the selectivity of LCI699 for *CYP11B2* over *CYP11B1* is highly dose dependent, resulting in a relatively narrow therapeutic window (**Table 2**): while relative selectivity can be maintained at low doses, pronounced off-target inhibition of *CYP11B1* emerges at doses in the range of 1–3 mg and above. *In vitro* assays indicate a selectivity ratio of only approximately 3.6 (IC50: 0.7 nM vs. 2.5 nM), with cross-species selectivity remaining within a 3–5-fold range; however, inhibitory potency exhibits marked interspecies differences (human > primate > rodent), underscoring the strong dependence of pharmacodynamic predictions on human enzyme models (132). With increasing doses, measurable effects on cortisol synthesis become evident, including suppression of both basal and adrenocorticotropic hormone (ACTH)–stimulated cortisol secretion, thereby constraining the clinically accessible dosing range (132–137).

Studies in patients with resistant hypertension and PA further indicate that, although LCI699 dose-dependently suppresses aldosterone production, its antihypertensive efficacy is inconsistent and generally inferior to that of MRAs (136, 137). Collectively, these observations suggest that the potential value of LCI699 may reside primarily in mitigating aldosterone-mediated target organ injury, rather than in functioning as a superior antihypertensive agent.

Notably, owing to its potent inhibition of 11β-hydroxylase at higher doses, LCI699 (osilodrostat) has been repositioned from an antihypertensive candidate to a therapeutic agent for Cushing’s syndrome. Recent studies demonstrate that it can rapidly normalize and maintain urinary free cortisol levels, with approximately 60–70% of patients achieving sustained biochemical control over mid- to long-term follow-up (138, 139). In parallel, its indication has been expanded to encompass patients with endogenous Cushing’s syndrome who are not candidates for surgery or who experience postoperative recurrence (139). However, the safety profile is dominated by hypocortisolism, glucocorticoid withdrawal–like symptoms, and elevations in steroid precursors, and a degree of persistent adrenal suppression may occur after treatment discontinuation, necessitating close monitoring and individualized dose titration (138, 139).

### 6.3 Second-generation ASIs: clinical breakthroughs driven by enhanced selectivity

Guided by insights into the structural differences between the crystal conformations of *CYP11B2* and *CYP11B1* and subsequent molecular optimization, next-generation nonsteroidal ASIs—such as baxdrostat and lorundrostat—have achieved markedly enhanced enzyme selectivity (**Table 2**), with inhibitory selectivity for *CYP11B2* reaching approximately 100:1 (140) and 374:1 (141), respectively, thereby conferring improved *in vivo* target specificity and a substantially reduced adverse-event profile (142, 143). In populations with resistant hypertension, both agents have demonstrated robust blood pressure–lowering efficacy, offering a new therapeutic option beyond spironolactone for patients with difficult-to-control or treatment-resistant hypertension.

#### 6.3.1 Baxdrostat

The phase II BrigHTN study, completed in 2022, demonstrated that the addition of baxdrostat to standard antihypertensive therapy further reduced blood pressure with an acceptable safety profile (144). The efficacy of baxdrostat was further confirmed in the phase III BaxHTN trial reported in 2025 (143): specifically, the 2-mg baxdrostat group achieved a mean systolic blood pressure reduction of approximately −15.7 mmHg, compared with −5.8 mmHg in the placebo group, a difference that reached statistical significance (143). Baxdrostat exhibited a favorable safety profile, with a low incidence of serious adverse events comparable to placebo. The primary safety signals were dose-dependent hyperkalemia and mild to moderate hyponatremia; however, rates requiring clinical intervention or treatment discontinuation were low, and no evidence of cortisol deficiency or other impairments in steroidogenesis was observed (143).

In PA, the 2025 SPARK phase II study (n = 15) showed that baxdrostat significantly reduced both plasma and urinary aldosterone levels, ameliorated hypokalemia, and produced meaningful blood pressure reductions, thereby suggesting potential utility in patients with bilateral PA or those who are not surgical candidates (145). However, the limited sample size and absence of a control group, together with signals of hyperkalemia and reversible declines in estimated glomerular filtration rate (eGFR) at higher doses (8 mg), warrant cautious interpretation of both efficacy and safety. Larger, dedicated PA trials, such as the ongoing Bax PA study (NCT07007793), have now been initiated and are expected to address the current evidence gap for pharmacological management of bilateral PA.

#### 6.3.2 Lorundrostat

By contrast, lorundrostat has also demonstrated compelling clinical performance in uncontrolled hypertension. In the 2023 Target-HTN phase II study, an 8-week course of lorundrostat 50 mg daily reduced systolic blood pressure by approximately 13.2 mmHg from baseline, compared with a 4.1 mmHg reduction in the placebo group, yielding a between-group difference of −9.6 mmHg (90% CI, −15.8 to −3.4; P = 0.01); the 100-mg dose produced a comparable magnitude of blood pressure reduction (difference of approximately −7.8 mmHg) (142). No reductions in cortisol levels were observed across dose groups, further supporting the high selectivity of lorundrostat for *CYP11B2* (142).

This efficacy was further corroborated in the large, multicenter Launch-HTN trial reported in 2025. Among approximately 1,083 patients with uncontrolled or resistant hypertension, 6 weeks of treatment with lorundrostat 50 mg resulted in a mean systolic blood pressure reduction of 16.9 mmHg, significantly greater than the 7.9 mmHg observed in the placebo group (between-group difference of −9.1 mmHg; P < 0.001) (146). During the study period, the incidence of adverse events was comparable to that of placebo; hyperkalemia, hyponatremia, and mild-to-moderate declines in renal function were infrequent, and approximately 0.5% of patients discontinued treatment due to electrolyte abnormalities, with no cases of severe renal failure observed (146).

#### 6.3.3 Summary

Overall, pivotal trials such as BrigHTN, Target-HTN, and Launch-HTN have established a solid evidence base for the antihypertensive efficacy and safety of second-generation ASIs, offering new therapeutic options for patients with resistant hypertension and PA. Nevertheless, although upstream suppression of aldosterone synthesis is theoretically poised to confer potential cardiorenal benefits beyond those achieved with conventional MRAs, ASIs may still elicit compensatory increases in renin and angiotensin II. Whether the observed short-term improvements in blood pressure will ultimately translate into definitive cardiovascular and renal outcome benefits remains to be determined by ongoing long-term outcome trials, including global phase III programs such as BaxAsia and Bax24 (147, 148). Notably, most existing *in vivo* studies have focused primarily on pharmacokinetic profiles, endocrine selectivity, and blood pressure–lowering effects, whereas direct mechanistic evidence with immuno-inflammatory endpoints—such as inflammatory cell infiltration and activation of the NLRP3/IL-1β signaling axis—remains relatively limited. Given the central role of inflammation and organ remodeling in the residual cardiovascular risk associated with PA, systematic evaluation of the effects of second-generation ASIs on these inflammatory pathways in standardized cardiovascular and renal injury models will be critical for clarifying their potential organ-protective value.

### 6.4 Emerging perspectives for aldosterone synthase inhibitors

Given the pivotal role of aldosterone in cardiovascular remodeling, tubulointerstitial fibrosis, and inflammatory responses, the clinical application of ASIs is progressively expanding beyond blood pressure control toward integrated management of cardiorenal comorbidities. On one hand, evidence from randomized clinical trials indicates that the ASI BI 690517 can significantly reduce the urinary albumin-to-creatinine ratio (UACR) by approximately 20–40% in patients with proteinuric chronic kidney disease, while maintaining a favorable tolerability profile (149–151). These findings suggest that ASIs may confer direct renoprotective effects beyond blood pressure control and provide a foundation for larger, long-term cardiorenal outcome studies, such as EASi-KIDNEY (151). On the other hand, in heart failure—particularly heart failure with preserved ejection fraction (HFpEF)—aldosterone-driven myocardial fibrosis and inflammation are increasingly recognized as central pathological substrates (152–154). Accordingly, ASIs are increasingly viewed as potential “multi-pathway convergence” nodes for therapeutic intervention. Several ongoing trials are now exploring the addition of ASIs on top of renin–angiotensin system (RAS) inhibitors and sodium–glucose cotransporter 2 (SGLT2) inhibitors, with the aim of achieving further reductions in proteinuria and delaying the progression of cardiorenal injury through multi-target combination strategies (150). In addition, individualized dosing strategies guided by plasma renin activity (PRA) or obesity-associated high-aldosterone phenotypes further underscore the potential of ASIs within a precision medicine framework.

Overall, ASIs have emerged as a novel class of endocrine and cardiovascular interventions, expanding from resistant hypertension toward a broader spectrum of aldosterone-related disorders, including PA, chronic kidney disease, and heart failure. Although current evidence is largely derived from short- to mid-term studies and definitive benefits on hard cardiovascular endpoints remain to be established, their well-defined mechanisms of action, high enzymatic selectivity, and relatively manageable safety profiles have laid the groundwork for large-scale trials with cardiorenal outcomes as primary endpoints. As phase III results continue to emerge, ASIs are poised to become an integral component of therapeutic strategies for resistant hypertension and PA, and to assume a more clearly defined role within the comprehensive management of cardiorenal–metabolic disease.

## 7 Integrated therapeutic strategies

### 7.1 Limitations of monotherapy

According to the 2025 Endocrine Society Clinical Practice Guidelines for PA, treatment should be individualized based on disease subtype and surgical eligibility: unilateral adrenalectomy is recommended for patients with lateralized PA confirmed by adrenal venous sampling, whereas long-term therapy based on MRAs is advised for those with bilateral adrenal disease, indeterminate lateralization, or who are not candidates for, or decline, surgery (155).

However, accumulating evidence indicates that MRA monotherapy does not fully eliminate the residual cardiovascular risk in patients with PA (41, 126). Even when blood pressure and serum potassium are adequately controlled, cardiovascular injury driven by persistent inflammatory responses, oxidative stress, and structural remodeling of target organs may persist, highlighting the intrinsic mechanistic limitations of single-pathway intervention.

As discussed in the preceding section on ASIs, recent advances in their application in PA have been systematically reviewed. Although upstream suppression of aldosterone biosynthesis is, in principle, capable of attenuating both its genomic and non-genomic actions, the role of ASIs as monotherapy remains uncertain. To date, there is insufficient evidence to demonstrate that long-term aldosterone suppression can fully reverse established myocardial fibrosis and vascular remodeling, or that it confers definitive benefits on hard cardiovascular endpoints. Moreover, even when aldosterone levels are substantially reduced, components of immune and inflammatory dysregulation may become partially self-sustaining and therefore incompletely responsive to hormonal load reduction alone.

Accordingly, while ASIs represent an important addition to the therapeutic armamentarium for PA, their use as monotherapy is unlikely to fully abrogate residual cardiovascular risk. These considerations support the need, within the framework of current guideline-recommended pathways, to integrate mechanistically complementary interventions that target aldosterone biosynthesis, receptor activation, and downstream inflammatory and remodeling pathways, thereby establishing a multi-pathway, synergistic treatment strategy aimed at achieving more comprehensive and durable cardiovascular risk reduction in PA.

Beyond the mechanistic limitations of monotherapy, the clinical heterogeneity of PA may also influence the nature of residual inflammatory and cardiovascular risk after treatment. Patients with aldosterone-producing adenoma (APA) and those with bilateral adrenal hyperplasia (BAH) differ in aldosterone secretion patterns, disease chronicity, and primary treatment strategies; therefore, residual risk may not be entirely identical between surgically treated and medically treated patients. In biochemically cured APA after adrenalectomy, persistent risk may largely reflect the legacy effects of prior aldosterone excess and incompletely reversible cardiovascular remodeling. In contrast, in BAH treated with long-term MRA therapy, residual risk may additionally relate to incomplete MR blockade, persistent renin suppression, ongoing non-genomic aldosterone signaling, and challenges in sustained biochemical control. Supporting this view, a recent meta-analysis of 20 studies involving 16,927 patients found that treated PA remained associated with a higher incidence of major adverse cardiovascular events (MACE) than non-PA hypertension. In addition, adrenalectomy was associated with a lower MACE incidence than MRA therapy (2.00 vs 3.30 per 100 patient-years, P = 0.017) (156). These findings suggest that residual risk in PA should be interpreted in a subtype- and treatment-specific manner, with implications for individualized follow-up and therapeutic intensification.

### 7.2 Rationale for combination and sequential therapeutic strategies

Given the limitations of single-modality approaches, integrating multi-target interventions at the mechanistic level to construct combination or sequential therapeutic strategies is supported by a clear theoretical rationale.

#### 7.2.1 Combined or sequential use of MR antagonists and ASIs

MRAs and ASIs act at distinct nodes of the aldosterone–MR axis: the former block receptor-level signal transduction, whereas the latter suppress aldosterone biosynthesis. In combination, they are theoretically capable of achieving an “upstream–downstream dual blockade” of aldosterone’s pathogenic effects, thereby reducing aldosterone escape and attenuating the persistent activation of non-genomic signaling. Clinical studies further indicate that, because ASIs do not directly interact with steroid hormone receptors, their adverse-effect profiles largely lack the sex hormone–related side effects commonly associated with spironolactone, conferring comparatively better overall tolerability (142, 146).

Notably, the effectiveness of MRA therapy depends not only on blood pressure control, but more critically on whether it succeeds in “un-suppressing” renin from its inhibited state (41, 157). Persistent renin suppression has been associated with an increased risk of adverse cardiovascular outcomes (89). Consistent with these findings, Hundemer et al. further demonstrated that PA patients whose renin remained suppressed after MRA therapy (PRA < 1 μg/L/h) had a significantly higher risk of cardiovascular events, whereas those in whom renin increased to ≥ 1 μg/L/h exhibited risk levels comparable to individuals with essential hypertension (41). In addition, Köhler et al. reported that left ventricular mass index (LVMI) serves as a key marker of myocardial reverse remodeling in PA, and that persistent renin suppression limits the reduction in LVMI and the extent of cardiac reverse remodeling (157). Collectively, these studies indicate that the therapeutic goal of MRA treatment in PA should extend beyond achieving target blood pressure, and instead incorporate the transition of renin from a suppressed to an elevated state as a physiological marker of adequate MR blockade. Accordingly, the renin response may serve as a practical guide for MRA dose titration to optimize cardiovascular protection.

Accordingly, in PA patients with persistently suppressed renin, it may be theoretically appropriate to intensify inhibition of the aldosterone–MR axis—either by up-titrating MRA doses or by combining MRAs with ASIs—with the goal of achieving renin “de-suppression” as a therapeutic target, thereby potentially reducing residual cardiovascular risk.

It should be noted that, despite their distinct targets, both ASIs and MRAs can increase the risk of hyperkalemia, and prior ASI clinical trials have likewise reported dose-dependent, reversible elevations in serum potassium (142, 146). In addition, combined blockade of aldosterone synthesis and MR signaling may increase the risk of acute kidney injury (AKI), highlighting the need for careful monitoring of electrolytes and renal function when such strategies are considered, particularly in patients with CKD, older age, or concomitant RAS blockade. At present, this strategy remains largely at the level of theoretical rationale and early clinical exploration, with insufficient evidence from large-scale studies to support its routine use. Accordingly, outside of specific research settings or selected cases of refractory disease, the combined use of ASIs and MRAs is not recommended in routine clinical practice.

#### 7.2.2 Theoretical basis for dual-pathway modulation of MR and GPER

Under aldosterone stimulation, GPER and MR do not operate as independent entities but instead form an interconnected signaling network that jointly regulates vascular tone, immune–inflammatory responses, and cardiovascular remodeling. Aldosterone exerts its actions through MR-mediated genomic effects on one hand, and through GPER-driven rapid non-genomic signaling on the other, with these parallel pathways acting synergistically to amplify proinflammatory and injurious responses. Experimental studies indicate that even when MR is pharmacologically blocked, GPER-associated inflammatory signaling and abnormalities in blood pressure regulation can persist, suggesting that this pathway may contribute to the residual cardiovascular risk observed in patients with PA following MR-based therapy (114, 115).

However, the functional heterogeneity of GPER across different tissues, disease stages, and ligand contexts, as well as its cooperative or antagonistic interactions with MR, remains incompletely understood. Accordingly, the future development of safe and effective GPER-targeted agents may, in theory, enable combination therapy with MR antagonists to concurrently modulate both the genomic and non-genomic actions of aldosterone—suppressing MR-mediated transcriptional pathogenic effects while blocking GPER-associated rapid proinflammatory signaling—thereby more effectively attenuating downstream pathological pathways and achieving more comprehensive cardiovascular protection.

#### 7.2.3 Potential value of combining MR antagonists with anti-inflammatory interventions

A substantial body of experimental and clinical evidence has established inflammation as a central driver of cardiovascular injury associated with PA (35). Patients with PA typically exhibit elevations in multiple proinflammatory biomarkers, including TNF-α, IL-6, and high-sensitivity C-reactive protein (hsCRP) (53, 158, 159). Notably, even after treatment with MR antagonists, oxidative stress and inflammatory activity may persist to some extent due to ongoing non-genomic actions of aldosterone and sustained activation of downstream signaling pathways, indicating that inflammatory cascades are not necessarily fully suppressed by MR blockade alone.

In populations characterized by cardiovascular–kidney–metabolic syndrome (CKM), exemplified by type 2 diabetes, residual cardiovascular risk driven by chronic low-grade inflammation commonly persists even when traditional cardiovascular risk factors are optimally controlled (160). Interventions targeting this residual inflammatory risk have demonstrated clinical benefit; for example, low-dose colchicine reduces the incidence of cardiovascular events, and anti-inflammatory strategies directed at the IL-6 pathway significantly lower inflammatory burden with the potential for additional risk reduction (160). By extension, in PA patients with a pronounced inflammatory phenotype, the addition of anti-inflammatory strategies to standard MRA-based therapy may further reduce the risk of cardiovascular events. This concept provides a new research direction for the development of integrated therapeutic approaches in PA.

## 8 Future perspectives and research directions

Despite a strong mechanistic rationale, the clinical value of integrated therapeutic strategies in PA remains to be established. Future research should prioritize:

- prospective studies with cardiovascular hard endpoints to determine whether combination or sequential strategies provide incremental benefit over monotherapy;
- validation of renin status, inflammatory markers, and fibrosis-related biomarkers to enable phenotype-guided and biomarker-driven management; and
- further clarification of non-classical aldosterone pathways, including GPER signaling, and translation of related targeted therapies to reduce residual cardiovascular risk beyond MR blockade.

## 9 Conclusion

PA is increasingly recognized as a disorder in which excess aldosterone promotes cardiovascular injury through mechanisms that extend beyond blood pressure elevation alone. A growing body of experimental and clinical evidence supports the concept that persistent inflammation represents a key driver of the residual cardiovascular risk observed in PA, even among patients achieving adequate blood pressure control. Excess aldosterone activates both innate and adaptive immune responses, including macrophages, T lymphocytes, inflammasome pathways such as NLRP3, and pro-inflammatory cytokines such as interleukin-6 (IL-6), thereby contributing to myocardial fibrosis, endothelial dysfunction, and adverse cardiovascular remodeling. Conventional MRAs attenuate these processes only partially, leaving a clinically meaningful inflammatory burden.

Recent therapeutic developments provide new opportunities to address this unmet need. Nonsteroidal MRAs, including finerenone and esaxerenone, offer improved receptor selectivity and tolerability and have demonstrated favorable effects on inflammation and end-organ protection that appear to extend beyond blood pressure lowering. In parallel, ASIs, such as baxdrostat and lorundrostat, represent an upstream approach that directly suppresses aldosterone biosynthesis. Early clinical studies suggest that ASIs can effectively reduce aldosterone levels, correct hypokalemia, and improve blood pressure control, particularly in patients with bilateral PA or those unsuitable for surgical intervention. Nevertheless, concerns regarding hyperkalemia and renal function highlight the need for larger, controlled trials specifically designed for PA populations.

Beyond RAAS-directed therapies, increasing attention has focused on downstream inflammatory pathways as potential therapeutic targets. IL-6 signaling and the NLRP3 inflammasome/IL-1β axis have emerged as important mediators of aldosterone-induced cardiovascular injury in experimental models, suggesting that targeted anti-inflammatory strategies may complement aldosterone blockade by addressing non-hemodynamic mechanisms of organ damage. In addition, GPER signaling has been implicated in aldosterone-related inflammation and vascular dysfunction, representing a novel but complex target that warrants further mechanistic and translational investigation.

Despite these advances, several challenges remain. Validated biomarkers to identify ongoing inflammation and residual cardiovascular risk in PA are lacking, and robust evidence linking emerging therapies to long-term cardiovascular and renal outcomes is still limited. Future studies should focus on biomarker development, optimization of therapeutic combinations and sequencing, and rigorous evaluation of clinical outcomes in PA-specific trials. Collectively, an integrated treatment strategy that combines aldosterone suppression with inflammation-oriented and precision-based approaches may offer a more comprehensive framework for reducing the cardiovascular burden associated with PA.
